# Prevalence of musculoskeletal symptoms among administrative workers at a teaching hospital in the state of Espírito Santo, Brazil

**DOI:** 10.47626/1679-4435-2021-658

**Published:** 2021-12-30

**Authors:** Mariana Bomfim Natali, Marcela Cangussu Barbalho-Moulim

**Affiliations:** 1 Departamento de Educação Integrada em Saúde, Universidade Federal do Espírito Santo, Vitória, Espírito Santo, Brazil.

**Keywords:** epidemiology, cumulative trauma disorders, occupational health, ergonomics, epidemiologia, transtornos traumáticos cumulativos, saúde do trabalhador, ergonomia

## Abstract

**Introduction:**

Musculoskeletal symptoms can affect workers’ quality of life and work productivity, leading to sick leave and company losses.

**Objectives:**

To identify the prevalence of musculoskeletal symptoms among administrative workers at a hospital in the state of Espírito Santo, the most frequent complaints, and the associated personal and occupational factors.

**Methods:**

This is an observational, cross-sectional study. Data were collected using an evaluation form with personal data and questions related to work conditions, the Nordic Musculoskeletal Questionnaire, which aims to identify musculoskeletal complaints in nine parts of the body, and the Visual Analog Scale.

**Results:**

Fifty participants were assessed, of whom 52% were women. The majority was aged 31 to 40 years. Approximately 92% of workers had some musculoskeletal complaint, and 6% had been on sick leave. Regarding pain location, the spine was the most commonly affected region, especially the lumbar spine, followed by the upper extremities (especially shoulders and fists/hands). Only 40% of workers had received ergonomic guidance, and 54% adhered to workplace exercise. In addition, 85% of those who received physical therapy reported pain improvement.

**Conclusions:**

Most administrative workers (92%) had complaints of musculoskeletal symptoms, predominantly in the lumbar spine.

## Resumo

**Introdução:**

Sintomas osteomioarticulares podem afetar a qualidade de vida do indivíduo e a produtividade no trabalho, podendo levar ao afastamento e a prejuízos à empresa.

**Objetivos:**

Conhecer a prevalência dos sintomas osteomioarticulares em trabalhadores do setor administrativo de um hospital do Espírito Santo, bem como as queixas mais frequentes e os fatores pessoais e profissionais associados.

**Métodos:**

Tratou-se de um estudo transversal, observacional. Para a coleta de dados, foi utilizado um formulário de avaliação que continha dados pessoais e perguntas relacionadas à condição de trabalho, além do Questionário Nórdico Musculoesquelético, que tem como objetivo identificar queixas osteomioarticulares em nove partes do corpo, e da Escala Visual Analógica.

**Resultados:**

Foram avaliados 50 voluntários, 52% do sexo feminino, sendo a maior parte com idade entre 31 e 40 anos. Cerca de 92% dos trabalhadores apresentaram alguma queixa de sintomas osteomioarticulares, e 6% já precisaram se afastar do trabalho. Com relação ao local de dor, a principal região citada foi a coluna, sendo a lombar a mais afetada, seguida da região dos membros superiores, principalmente ombros e punhos/mãos. Apenas 40% dos trabalhadores receberam orientação sobre ergonomia, e 54% realizavam ginástica laboral no trabalho. Ainda, 85% daqueles que realizaram fisioterapia obtiveram melhora das queixas.

**Conclusões:**

Foi evidenciado que uma grande parte (92%) dos trabalhadores do setor administrativo apresentavam queixas de sintomas osteomioarticulares, sendo a coluna lombar a mais afetada.

## INTRODUCTION

Work-related musculoskeletal disorders (WMSDs), which are linked to the musculoskeletal system, are caused by work-related activities and can lead to day- or month-long sick leave.^1^ These disorders may cause inflammation and degeneration, impairing major structures such as muscles, nerves, tendons, and joints, leading to pain and limited work and social involvement.^2^ WMSDs are characterized by symptoms that may occur concomitantly or not, such as pain, paresthesia, feelings of heaviness, and fatigue.^3^

According to data from the United Nations (UN) published in 2013, 160 million people worldwide are affected by non-lethal work-related diseases.^4^ The onset of musculoskeletal symptoms has been increasing globally, accounting for the second leading cause of sick leave in Brazil. Consequently, this leads to decreased productivity, which considerably impacts the economy at the business, government, and societal levels.^1,2^ Industry, sales, food, transport, and cleaning/domestic workers have the highest prevalence of musculoskeletal symptoms.^5^

Preventive measures aimed at improving occupational health and quality of life, such as ergonomics-related physical therapy, can help reduce risk factors for work-related disorders and musculoskeletal complaints.^1^ Ergonomics aims to reduce risk factors associated with the workplace, whereas physical therapy, together with preventive techniques, helps to prevent the onset of pain and physical discomfort.^1^

Identifying the prevalence of musculoskeletal disorders among administrative workers at a hospital in Espírito Santo is of great relevance, as these disorders may affect workers’ quality of life and work productivity, which can lead to sick leave and, consequently, to company losses. In addition, in-depth knowledge of the issue can lead to the adoption of strategies to prevent these complaints, such as physical therapy, improving workers’ health and productivity.

Therefore, the general objectives of this study were to identify the prevalence of musculoskeletal symptoms among administrative workers at a hospital in Espírito Santo, identify the most frequent complaints, and correlate findings with occupational and personal factors.

## METHODS

This observational, cross-sectional study was conducted in the city of Vitória, state of Espírito Santo, Brazil, from November to December 2019. The research project was approved by the institutional research ethics committee (approval number 3.698.616).

The study population consisted of administrative workers, clerks, and assistants at a hospital in Espírito Santo. A total of 50 workers from the administration, tender, accounting, invoicing, and IT departments, from both sexes, aged 20 to 60 years, were included. Those with more than one employment relationship or who performed other work activities were excluded.

Visits were made to each aforementioned department. All participants received detailed information on the research and signed the informed consent form. Subsequently, they were invited to complete the questionnaires.

Data were collected using an evaluation form with personal data and questions related to work conditions, ergonomics, and physical therapy. Then, the Nordic Musculoskeletal Questionnaire (NMQ) was applied,^6^ which aims to identify musculoskeletal discomfort in nine parts of the body in the last 12 months and last 7 days and activity limitations in the last 12 months. The Visual Analog Scale (VAS) was also applied. It is an easily understandable scale used to assess pain, with scores ranging from 0 to 10, where 0 means “no pain” and 10 means “worst pain.”

Study variables were expressed as absolute frequencies and percentages. Pearson’s chi-square test was used to investigate the association of demographic variables (sex, age, weight, and body mass index [BMI]) and time of employment with the frequency of musculoskeletal symptom complaints. The significance level was set at 5%. Data analysis was performed using BioStat 5.0.

## RESULTS

The sample consisted of 50 administrative workers at a hospital in Espírito Santo, aged 20 to 60 years, of whom 52% were women. Most participants were classified as adults aged 31 to 40 years (46%), with less than 5 years on the job (86%) and a mean workload of 41.30 ± 3.39 hours per week. Additional information is provided in Table 1.

**Table 1:** Demographic profile of the study population, Cassiano Antônio de Moraes Teaching Hospital, Vitória, state of Espírito Santo, Brazil, 2020

Variable	Absolute value (n = 50)	Percentage
Age (years)		
Between 20 and 30	13	26
Between 31 and 40	23	46
Between 41 and 50	7	14
> 51	7	14
Sex		
Female	26	52
Male	24	48
Height (m), mean ± SD	1.70 ± 0.82	-
Weight (kg), mean ± SD	75.92 ± 17.66	-
BMI (kg/m^2^), mean ± SD	26.15 ± 4.93	-
Dominant limb (R/L)	46/4	92/8
Time of employment (years)		
Up to 5	43	86
Between 6 and 10	2	4
Between 11 and 20	3	6
> 20	2	4
Weekly workload (hours), mean ± SD	41.30 ± 3.39	-
Number of complaints per sex		
Female	122	-
Male	118	-

BMI = body mass index; R/L = right/left; SD = standard deviation.

**Figure 1:**
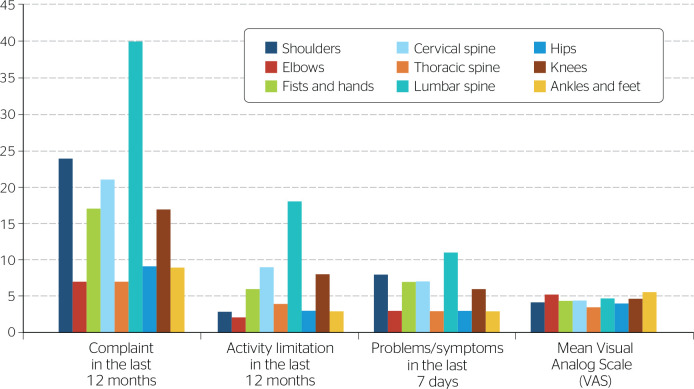
Answers provided in the Nordic Musculoskeletal Questionnaire (NMQ).

Of 50 participants, 46 (92%) reported musculoskeletal complaints in the NMQ. Most participants reported mild to moderate pain; VAS scores ranged from 4 to 6, with a mean pain score of 4.49 ± 0.63. Regarding pain location, the spine was the most affected region, with 68 complaints in the last 12 months and 21 in the last 7 days, predominantly in the lumbar spine. The upper extremities were the second most affected region, with 48 complaints in the last 12 months and 18 complaints in the last 7 days, predominantly in the shoulder joints. In addition, 35 participants complained of pain in the lower extremities in the last 12 months and 12 in the last 7 days, predominantly in the knees. Additional information is provided in Figure 1.

When participants were asked about having received ergonomic guidance, 40% reported having received it, of whom 65% reported following the recommendations. In addition, 54% reported the presence of exercise programs in the workplace. A total of 26% participants had received physical therapy due to musculoskeletal complaints, of whom 85% reported pain improvement. However, 6% had been on sick leave due to musculoskeletal symptoms. Additional information is provided in Table 2.

**Table 2:** Answers provided in the general data collection questionnaire developed by the authors, Vitória, state of Espírito Santo, Brazil, 2020

Questions	Yes	
	n	%
1. Have you ever received ergonomic guidance from a physical therapist?	20	40
2. If yes, do you follow the recommendations?	13	65
3. Do you receive regular guidance for musculoskeletal symptom prevention?	7	14
4. Is there an exercise program in your workplace?	27	54
5. Do you take breaks during the workday?	31	62
6. Have you ever felt/do you feel any pain that might be related to work?	29	58
7. Have you ever received physical therapy?	13	26
8. Was the treatment successful?	11	85
9. Are you currently receiving physical therapy to improve musculoskeletal symptoms?	1	2
10. Have you ever been on sick leave due to musculoskeletal symptoms?	3	6

After correlation analysis using Pearson’s test, the number of complaints per participant was not significantly correlated with time of employment, sex, age, weight, or BMI.

## DISCUSSION

The sample consisted of 50 administrative workers at a hospital in the state of Espírito Santo, of whom 52% were women. Most participants were adults aged 31 to 40 years (46%), with less than 5 years on the job (86%) and a mean workload of 41.30 ± 3.39 hours. According to the answers provided in the NMQ, pain was predominant in the spine, especially in the lumbar segment, followed by the upper extremities, especially in the shoulder joints. Only 40% of participants had received ergonomic guidance, of whom 65% reported following the recommendations. In addition, 54% reported the presence of a workplace exercise program, and 6% had been on sick leave due to musculoskeletal disorders.

Regarding age group, previous studies found that most administrative workers were aged 30 to 50 years, with a workload of 40 hours per week. The number of complaints was associated with age, meaning that pain may result from aging-related degradation, and may be associated with time of employment and changes in the workplace.^7,8^ However, in the present study, the number of complaints was not significantly correlated with time of employment, sex, age, weight, or BMI.

Administrative workers complained mainly of pain in the neck, shoulders, fists/hands, and lumber spine, which suggests a possible association between occupational characteristics and pain in these regions.^9,10^ Importantly, most activities performed by these workers are office-related, meaning that they spend most of the time sitting in front of the computer. The psychological pressure to be more productive in the workplace is another factor related to the onset of these complaints, and it could also be associated with the onset of cervical and lumbar spine pain.^11^

Given the elevated number of complaints, as previously demonstrated, the literature shows the importance of ergonomic guidance and exercise programs in the workplace. Not following these recommendations, together with individual factors, inadequate strength, poor posture, mental health, prolonged static posture, highly repetitive work, use of tools, psychosocial factors, not taking breaks, shift work, and low job security, may be associated with the onset of musculoskeletal disorders.^12,13^ An alternative to help prevent and reduce musculoskeletal pain is adherence to workplace exercise, including muscle strengthening, stretching, and relaxation exercises for this purpose.^14^

In addition to physical factors, psychosocial factors have also been cited as a trigger for the onset of musculoskeletal disorders. These factors can be widely evaluated using the International Classification of Functioning, Disability, and Health (ICF), which includes the process of illness and the requirements for a healthy presence at work. Some ICF definitions, such as fine hand use and hand and arm use, will affect routine tasks and may result in the onset of psychosocial disorders that are often also related to musculoskeletal disorders.^7^

All the aforementioned factors are also related to the increased frequency of sick leave. Shoulder lesions, synovitis and tenosynovitis, and rotator cuff syndrome are the leading causes of sick leave and accounted for approximately 50% of temporary disability payments between 2004 and 2016 in Brazil.^15^ A study showed that musculoskeletal disorders were the second leading cause of morbidity among Brazilians with employment relationship, and that 56% of administrative workers had been on sick leave due to musculoskeletal disorders.^16^ Comparing these data with the data from our study, the prevalence of sick leave was low, affecting only 6% of our participants. This may be explained by the fact that our study population was relatively young and had a short time of employment.

The joint work between companies and regional occupational health reference centers (*centros de referência em saúde do trabalhador*, CEREST) is extremely important to promote health education actions for workers and to prevent musculoskeletal disorders. These actions would include a combined intervention program consisting of ergonomics, explanatory brochures, and changes in the workplace.^17^

## CONCLUSIONS

The analysis of the results showed that most administrative workers (92%) at a hospital in the state of Espírito Santo complained of musculoskeletal symptoms. The spine was the most commonly affected region, especially the lumbar spine, followed by the upper extremities (shoulders, fists, and hands). Even though the study population consisted mostly of adults (aged 31 to 40 years) with a short time of employment (85% were < 5 years on the job), some workers had been on sick leave due to musculoskeletal complaints. The number of complaints was not significantly correlated with time of employment, sex, age, weight, or BMI. Given the high prevalence of complaints among participants, further research is required to assess the effectiveness of preventive measures on these symptoms, such as the distribution of educational brochures on ergonomics and exercise, preventive physical therapy, and expanding workplace exercise to all workers, thus contributing to improved quality of life and productivity.
